# Correction to: Two-stage framework for optic disc localization and glaucoma classification in retinal fundus images using deep learning

**DOI:** 10.1186/s12911-019-0876-y

**Published:** 2019-07-31

**Authors:** Muhammad Naseer Bajwa, Muhammad Imran Malik, Shoaib Ahmed Siddiqui, Andreas Dengel, Faisal Shafait, Wolfgang Neumeier, Sheraz Ahmed

**Affiliations:** 1 Fachbereich Informatik, Technische Universität Kaiserslautern, 67663 Kaiserslautern, Germany; 2 Deutsche Forschungszentrum für KünstlicheIntelligenz GmbH (DFKI), 67663 Kaiserslautern, Germany; 3 Deep Learning Laboratory, National Center of Artificial Intelligence, Islamabad, 46000 Pakistan; 4 School of Electrical Engineering and Computer Science (SEECS), National University of Sciences and Technology, H-12, Islamabad, 46000 Pakistan; 5 Ophthalmology Clinic, Rittersberg 9, 67657 Kaiserslautern, Germany

**Correction to: Bajwa et al. BMC Medical Informatics and Decision Making (2019) 19:136**

**https://doi.org/10.1186/s12911-019-0842-8**

Following publication of the original article [1], the authors noted a typesetting mistake in eqs. 1, 2 and 4. The equations are in the form of a fraction but the denominators of the fraction appeared beside the fraction. The equations are reproduced in this Correction article and the original article has been corrected.

1 $$ \mathrm{Precision}=\frac{\mathrm{TruePositives}}{\mathrm{TruePositives}+\mathrm{FalsePositives}} $$

2 $$ \mathrm{Recall}=\frac{\mathrm{TruePositives}}{\mathrm{TruePositives}+\mathrm{FalseNegatives}} $$

4 $$ \mathrm{Specificity}=\frac{\mathrm{TrueNegatives}}{\mathrm{TrueNegatives}+\mathrm{FalsePositives}} $$
